# Peri-operative care of elderly patients – an urgent need for change: a consensus statement to provide guidance for specialist and non-specialist anaesthetists

**DOI:** 10.1186/2047-0525-2-6

**Published:** 2013-03-27

**Authors:** Chris Dodds, Irwin Foo, Kerri Jones, Shiv Kumar Singh, Carl Waldmann

**Affiliations:** 1 James Cook University Hospital, Middleborough, UK; 2 Western General Hospital, Crewe Road South, Edinburgh, EH4 2XU, UK; 3 Torbay Hospital, Torquay, UK; 4 Royal Liverpool Hospital, Liverpool, UK; 5 Royal Berkshire Hospital, Reading, UK

## Aims and scope of the statement

Compared to younger, fitter patients, elderly patients are at a disproportionately greater risk of avoidable morbidity and mortality. In this statement, we aim to give guidance on the role of anaesthetists in optimising care of elderly patients by discussing principles relevant to the whole care pathway.

Elderly patients are a highly heterogeneous group, often frail with multiple comorbidities. Therefore, consideration throughout the care pathway must be given to elderly patients as individuals. We will emphasise that one of the best ways to optimise care during the peri-operative period is to apply the core principles that have already been successfully applied to well-managed day-case care. We will also emphasise how best practice in anaesthesia for elderly patients aligns with enhanced recovery.

## Overview

Anaesthetists are increasingly likely to encounter elderly patients in their everyday practice, given the rising numbers of elderly people in the UK. However, there is currently a lack of recent guidance for anaesthetists for this patient group. Therefore, in May 2010, a panel of experts convened to develop consensus on anaesthesia of elderly patients, in consultation with the Age Anaesthesia Association (AAA). All panel members are practicing clinicians with widespread experience in anaesthesia of elderly patients

### Changing patient demographic

In 2009 the percentage of the UK population aged 65 years and over was 16%. By 2034, this figure is projected to rise to 23%. The fastest population increase has been in those aged 85 and over; in 2009 there were 1.4 million people in the UK over the age of 85 years. This number is set to increase to 3.5 million by 2034 [1].

The estimated NHS expenditure per capita in England for 2011 is almost £985 for the 65 to 69 age group, rising to over £2,705 for the over 85 age group [2]. The cost to the NHS of hip fractures alone has been estimated at around £1.8 billion a year [3]. Falls in over 65 year olds cost the NHS an estimated £4.6 million a day [4].

An increasing proportion of the population is elderly; therefore, it is imperative that anaesthetists become familiar with managing elderly patients and that they understand the wider implications of their practice for the whole patient care pathway and for the wider NHS.

### The National Confidential Enquiry into Patient Outcome and Death (NCEPOD)

The 2001 NCEPOD report, *Changing the Way We Operate*, highlighted the strain the ageing population was having on clinical service provision [5]. Despite these warnings, the 2010 NCEPOD report, *Elective & Emergency Surgery in the Elderly: An Age Old Problem*, showed that there is still a long way to go to ensure good practice and appropriate care of elderly people in hospitals. The report cited that almost two-thirds of elderly patients received poor care and one in five had no pain assessment pre-operatively. In addition, pain was not assessed regularly post-operatively in almost a quarter of patients [3].

### Lack of guidance in this field

There is currently a lack of recent guidance for anaesthetists in the treatment and care of elderly patients. The latest guidance, *Anaesthesia and Peri-Operative Care of the Elderly*, was published by The Association of Anaesthetists of Great Britain and Ireland (AAGBI) in 2001 [6].

## Key issues in anaesthesia of the elderly

The key issues in anaesthesia of the elderly include the following:

•Designing a pathway capable of individualisation

•Principles of care for elderly patients

•Dynamic testing.

These will be discussed in turn.

## Designing a pathway capable of individualisation

### Elective setting

Elderly patient outcomes following surgery are highly dependent on the care they receive throughout their entire pathway. The design of the care pathway should begin as soon as the GP considers referring the patient for surgical opinion. If surgery is deemed necessary, the GP should start optimising the health of the patient, for example, by reviewing medication, providing a targeted exercise plan, providing dietary advice and advising on the cessation of smoking. Pre-assessment of the patient should provide an interface between general practice and surgery; this will be discussed again later.

Measures are already in place in the elective setting to improve the care pathway of elderly people undergoing surgery. It is imperative that such measures take into account the identification, assessment of severity and management of frailty of patients. One very good model of a pathway approach for elderly care is the proactive care of older people undergoing surgery (POPS) pathway [7]. This pathway involves multidisciplinary, pre-operative, comprehensive geriatric assessment. Implementation of the pathway yielded clear improvements; length of stay was reduced and there were fewer delayed discharges.

The report by the Improving Surgical Outcomes Group (ISOG), *Modernising Care for Patients Undergoing Major Surgery: Improving Patient Outcomes and Increasing Clinical Efficiency* (2005), stressed the need to establish pre-operative assessment at an early stage in the patient pathway, before surgery planning occurs, in order that decisions can be made about fitness for various procedures [8]. The report also emphasised that anaesthetists and critical care workers should be involved in the assessment of patients at an early stage [i.e. before the multidisciplinary team (MDT) meeting] to enable appropriate planning of surgery.

By the time any patient arrives for surgery, they should have undergone pre-assessment. Patients identified as needing optimisation should be reviewed against the criteria set out for their optimisation during pre-assessment.

### Emergency setting

Emergency surgery carries far greater risks than elective surgery in elderly patients; both morbidity and mortality are higher. This is often because patients undergoing emergency surgery are more likely to be acutely ill on admission and time for optimisation is likely to be short. In a study of 255 octogenarians receiving intensive care following major bowel surgery, mortality was twice as high in emergency cases in comparison to elective cases, at over 40% [9].

Unlike elective surgery, there is no routine, coherent pathway approach for care of the elderly in the emergency setting. However, it is vital that such a pathway is developed. This should include a rapid evaluation of how the patient can be optimised in advance of surgery, e.g. optimisation of pain and fluid therapy. This evaluation should be performed by an experienced clinician [3]. Optimisation of other factors, e.g. nutritional status and drug regimen, should be on-going as the patient progresses through the pathway [3] and should prompt referrals through the pathway to the enhanced recovery or discharge management team. Even though there is greater emphasis for the involvement of physicians from the department of medicine for the elderly (DME), input from anaesthetists is key to the optimisation process.

### Discharge management as a way to reduce readmission rates

Elderly patients undergoing surgery are at risk of a decline in physical and/or mental functioning, which may not have been resolved at the time of discharge. Without adequate discharge management planning, elderly patients are at greater risk of being re-admitted to hospital or of being prematurely admitted into long-term care. A recent Cochrane review suggested that hospital re-admission rates were significantly lower when discharge management planning was used compared with usual care [10]. Effective discharge management is essential to ensure smooth transition of elderly patients from hospital to home/community care without unplanned re-admission.

## Principles of care for elderly patients

The core principle that day care should be the default position for all elective surgery is as true for the elderly as for any other patient group (Modernisation Agency High Impact Change No. 1). In-patient care should be chosen only where there are specific indications. Well-managed day-case care, where patient outcomes are optimised, is built on core principles that are applicable for all patients. These principles should not just be applied to day-case care but should underpin the care of every patient and are enshrined in *enhanced recovery* pathways. Many of the principles of these pathways can also be adapted for emergency surgery.

Principles that can be translated to in-patient care pathways include:

•The adoption of a ‘Fitness for Referral’ strategy by primary care physicians for elective referrals

•Thorough pre-operative preparation in secondary/tertiary care including the assessment of cognitive dysfunction and the provision of excellent patient/carer information

•The institution of a shared decision-making process, which is particularly applicable to the elderly person to enable them to make a meaningful risk vs. benefit analysis of the intended procedure

•Early assessment of the risk of post-operative delirium, enabling processes to be put in place quickly to support and care for the patient with optimal effect

•The use of staff who have the necessary skills to treat elderly patients, including staff:

◦ Capable of performing a rapid assessment of cognition (see next point)

◦ With awareness of enhanced recovery principles and the ability to apply them

•Awareness of frailty and cognitive dysfunction

◦ Clinicians assessing the elderly should be aware that what most differentiates these patients from other patient groups is their frailty and increased risk of cognitive dysfunction; assessment of the latter should be routinely assessed pre-operatively since a patient who has cognitive impairment before surgery is highly likely to develop POCD.

◦ Knowledge of the patient’s pre-surgery cognitive state will help inform the rest of the patient’s treatment pathway. However, there is currently no training in assessment of cognition.

•Rapid mobilisation of the patient

◦ This principle has ramifications for several areas of the patient care pathway. A key assumption of enhanced recovery is that early ambulation translates into early discharge [11]. For anaesthetists, the principle has a direct impact on the patient immediately post-surgery and techniques should be tailored accordingly.

•Drug dosing

◦ Employment of anaesthetic techniques that lead to rapid recovery of the patient, including:

◦ The use of anaesthetic agents that give rapid recovery with minimal side effects

◦ Monitoring of intra-operative depth of anaesthesia

◦ Optimisation of peri-operative fluid management

◦ Appropriate pain management strategies to facilitate early mobilisation and oral nutrition

•Nearly all anaesthetic agents target the central nervous system (CNS), meaning any changes in the CNS due to the ageing process will have an impact on anaesthesia of the elderly. Furthermore, the distribution and elimination of anaesthetics show variable change with ageing. Owing to the heterogeneity of the elderly patient population, the dosing of anaesthetics in this patient group is subject to high levels of variation. This means that elderly patients are often at risk of under or overdosing, with the latter being more usual. However, dosing of anaesthetics is difficult to assess, apart from by clinical judgement.

•*Minimum alveolar concentration (MAC):* MAC decreases with increasing age by up to 40% [12]. Nomograms can be used to ensure over-dosing does not occur [13].

◦ Residual (0.1%) concentrations of volatile anaesthetic may remain detectable for between 33 to 71 h after administration [14].

•*Depth of anaesthesia monitoring:* Depth of anaesthesia monitoring, such as bispectral index or entropy monitoring, is a very useful tool in elderly patients as it might allow for better dose optimisation and improved outcomes. Several studies have shown an association between duration of inappropriate depth of anaesthesia and outcomes [15-18]. However, uncertainties remain as to whether depth of anaesthesia is a marker of functional reserve. Anaesthetic agents should be chosen that allow rapid titration and have limited context-sensitive changes in half-life.

## Dynamic testing

In line with enhanced recovery, the ISOG report (2005) stressed the requirement to implement new standards of care that incorporate improved pre-operative assessment, preparation and triage, intra-operative care and improved use of post-operative resources [8]. Since elderly patients are a highly heterogeneous group, static assessment methods are not adequate. Rather, a dynamic approach should be employed so that treatment can be individualised along the whole care pathway. Here, we give some examples of dynamic tests that can be used in elderly patients.

### Pre-operative testing

The Department of Health emphasises that an essential feature for enhanced recovery is the need for patients to be in the best possible condition for surgery [19]. Identification and treatment of pre-existing conditions should ideally be dealt with by the GP prior to referral or, at the latest, at the pre-operative assessment [19]. However, risk assessment may be particularly difficult in the elderly surgical population. Senior clinicians in surgery, anaesthesia and medicine need to be involved in the decision to operate on elderly patients [3,8].

### Pre-operative testing example: cardiopulmonary exercise (CPX) testing

Dynamic tests, such as CPX testing, are essential for the identification of at-risk patients undergoing surgery. Wilson *et al*. (2010) showed that routine measurement of the anaerobic threshold and elevated ventilatory equivalent for carbon dioxide (VE/VCO_2_) using CPX testing accurately identified the majority of high-risk patients undergoing non-vascular intra-abdominal surgery [20]. They found that the predictive benefit of CPX testing is just as apparent in those patients with no history of ischaemic heart disease or any other cardiac risk factors. Use of clinical risk factors alone only identified a relatively small proportion of at-risk patients, implying that clinical history cannot safely be used as a screening measure for selecting patients for CPX testing.

CPX testing has been advocated in the NCEPOD 2010 report. Most of the patients in the study underwent urgent or emergency surgery; therefore, CPX testing would not usually have been applicable. However, in those patients who had attended a pre-assessment clinic, very few had received formal assessment of cardiopulmonary reserve. The report concluded that CPX would have provided a more specific method of providing targeted management to those patients undergoing major surgery [3].

### Pre-operative testing example: frailty testing

Geriatric frailty is found in 20–30% of the elderly population over 75 years of age and increases with advancing age [21]. Frailty poses a unique clinical challenge, owing to its multifactorial nature [22]. It has been linked to increased risk of death, falls and institutionalisation [3,21]. The NCEPOD 2010 report identified that whereas frailty is recognised in the surgical setting of the elderly, it may not have been sufficiently factored into risk assessments and subsequent optimal planning of care.

The NCEPOD 2010 report noted that frailty is variously defined, but gives an operational definition, as per the Canadian Veterans Heart Study Definition of frailty, which states that frailty exists when the patient displays any three of the following [3,23]:

•Unintentional weight loss (at least 4 kg in last year)

•Self-reported exhaustion

•Weak grip strength

•Slow walking speed

•Low physical activity.

The NCEPOD study showed that the most common method of assessing frailty was by history and examination, and specific scoring systems were not cited. Indeed, there are no specific, validated, pre-operative tests for frailty. The ideal tests would be specific, simple, dynamic and quick to perform. Four examples of tests that could be included in an assessment of frailty are:

•Hand grip test

•Get up and go test

•Mini mental state examination

•Walking speed [24].

Once frailty has been identified in a patient its assessment and management require multidisciplinary input from surgeons, anaesthetists, nursing, rehabilitation, nutritionists and early involvement of Medicine for the Care of Older People [3,22].

### Cognitive dysfunction: the need for a simple, dynamic test

Testing for cognitive dysfunction in elderly patients poses particular difficulties owing to comorbidities and disabilities, such as the patient being blind or deaf. Although there are many general tests of cognitive dysfunction available, such as the Abbreviated Mental Test (AMT) and the Mini-Mental State Examination (MMSE), none are completely satisfactory for elderly surgical patients. Whilst we think testing of cognitive dysfunction is essential, we cannot advocate use of any particular test. However, the ideal test would be dynamic, quick and easy to perform.

### Intra-operative testing

Minimally invasive monitors that can be easily utilised intra-operatively include LIDCOplus™, LIDCOrapid™ and the CardioQ™ oesophageal Doppler monitor. Use of oesophageal Doppler monitors to optimise flow-related haemodynamic variables improves short-term outcomes in patients undergoing major abdominal surgery [25]. Currently, there is no targeted research on these techniques in the elderly.

### Post-operative testing – Delirium testing and management

The majority of elderly patients presenting for major surgery are at risk from delirium. Early identification of delirium post-surgery, i.e. in the recovery room, allows for more effective targeting for referral and treatment of patients than pre-operative identification. Identification of delirium should trigger the implementation of a management plan to optimise patient care. This should involve liaison with a geriatrician, psychiatrist, community psychiatric nurse, occupational therapist, GP or social services for further assessment, follow-up and/or social support.

### Outcomes of delirium

The prevalence of post-operative delirium in elderly patients ranges from 0% to 73%, depending on the study and type of surgery [26]. Post-operative delirium is a medical emergency, which can occur within hours of surgery and has the potential to last up to 7 days [27]. At least a quarter of elderly patients who develop delirium post-operatively may continue to have symptoms for up to 6 months after hospital discharge [28].

It is important to be aware that delirium also occurs where sedation is used in regional anaesthesia [29].

Recent guidelines from the National Institute for Health and Clinical Excellence (NICE) emphasise the serious consequences of delirium, including increased risk of dementia, death, increased length of stay in hospital and increased risk of new admission to long-term care [30]. Delirium may also be a marker for development of early post-operative cognitive dysfunction (POCD). The relationship between POCD and delirium has yet to be fully elucidated.

### The recovery room as a place for delirium testing

Delirium is often seen in the recovery room and is a good predictor of post-operative delirium on the ward [31,32]. Routine testing in the recovery room is an effective way of selectively identifying patients to receive post-operative care.

Any test for delirium in the recovery room should be quick and easy to perform by a range of healthcare professionals, including doctors and nurses. Rapid, early recovery of patients in the recovery room will aid the assessment process. The following have been used for delirium testing:

•The Confusion Assessment Method (CAM) [33,34]

•The CAM for the intensive care unit (CAM-ICU) [30,35]

•The Nursing Delirium Screening Scale (Nu-DESC) [31,36]

Owing to the potential for significant events identified post-operatively to cause long-term complications, GPs should be notified if patients develop acute delirium and/or cognitive dysfunction during the post-operative course and informed of the resulting MDT pathway.

### Post-operative assessment and management

Elderly patients suffer as much, if not more, pain after surgery than younger patients as they are likely to have to contend with the pain of stiff, arthritic joints and a greater incidence of chronic pain syndromes, as well as incisional/surgical handling pain.

Pain assessment protocols for younger patients do not readily reflect the severity of pain in the elderly. Differences in language, special senses (vision and hearing) and attitudes to pain make interpretation of the scoring more challenging. Attitudes of healthcare providers may limit the provision of effective analgesia, despite a pain score indicating inadequate pain control.

There is no single test/scoring scheme for elderly patients that can be recommended at the moment, but dynamic pain testing is essential in this age group, and specific scoring should be used in patients with identified cognitive dysfunction.

The development of pathways that assess and effectively manage acute pain throughout the peri-operative period is essential [37].

## Conclusions

The elderly surgical patient demands the highest level of care throughout their pathway from consideration of a surgical opinion to returning to their home.

This can only be delivered if significant changes are made as a matter of urgency. These changes include:

•The development of ‘fitness to referral’ pathways in primary care

•Effective individualised pre-operative assessment and optimisation

•Tailored surgical management to the overall clinical and functional state of the patient

•Careful choices on anaesthetic agents and techniques to maximise rapid recovery and effective pain control whilst avoiding excessive dosing

•Effective assessment in the recovery area of pain, cognitive function and physiological recovery, e.g. normothermia, fluid balance, oxygenation

•Discharge planning, across teams, which begins on referral and is reviewed during the entire pathway including return to home.

There are effective models that deliver parts of this pathway but no integrated whole as yet. This is a matter of great concern and one that needs urgent engagement and development.

## Abbreviations

AAA: Age anaesthesia association; AAGBI: Association of anaesthetists of great Britain and Ireland; AMT: Abbreviated mental test; CAM: Confusion Assessment method; CAM-ICU: Confusion assessment method for the intensive care unit; CNS: Central nervous system; CPX: Cardiopulmonary exercise; DME: Department of medicine for the elderly; ISOG: Improving surgical outcomes group; MAC: Minimum alveolar concentration; MDT: Multidisciplinary team; MMSE: Mini–mental state examination; NCEPOD: National confidential enquiry into patient outcome and death; NICE: National institute for health and clinical excellence; Nu-DESC: Nursing delirium screening scale; POCD: Post-operative cognitive dysfunction; POPS: Proactive care of older people undergoing surgery; VE/VCO2: Ventilatory equivalent for carbon dioxide

## Competing interests

In May 2010 Baxter convened a panel of experts to discuss the clinical applications of Suprane. This consensus document is the result of that meeting and consultation with the Age Anaesthesia Association (AAA). The authors have received payments from Baxter for consultancy work.

## Authors’ contributions

CD, IF and KJ were the main contributors to this consensus statement. All authors read and approved the final manuscript.
